# A rare case of melanoma arising in a nevus of the umbilicus in an adult patient

**DOI:** 10.1016/j.jdcr.2025.10.008

**Published:** 2025-10-11

**Authors:** Pamella Leybengrub, Samantha Ouellette, Rachel Manci, Tara Kaufmann

**Affiliations:** a Renaissance School of Medicine at Stony Brook University, Stony Brook, New York; b Stony Brook Dermatology, Stony Brook, New York

**Keywords:** melanoma, primary cutaneous melanoma, skin cancer, umbilical melanoma, umbilicus

## Introduction

Melanomas are malignant neoplasms that arise from melanocytes. Umbilical melanomas pose a significant diagnostic challenge due to their rare presentation. One study examining 112 umbilical tumors found only 4 of these lesions were diagnosed as malignant melanoma.^1^ Most malignant umbilical lesions are due to metastatic tumors from adjacent organs, and primary malignant tumors of the umbilical region much more rarely arise in clinical practice.^2^ Early detection and management is critical, as melanomas, when identified at an early stage, have a much more favorable prognosis. This case report presents a rare instance of melanoma arising from a nevus of the umbilicus in an adult patient, highlighting the importance of vigilant clinical assessment and timely intervention. By discussing the clinical features, diagnostic approach, and treatment of this case, this report aims to contribute to the limited body of knowledge on melanomas in this atypical location and to raise awareness about the potential for malignant transformation of nevi located in the umbilical region.

## Case report

**Fig 1:**
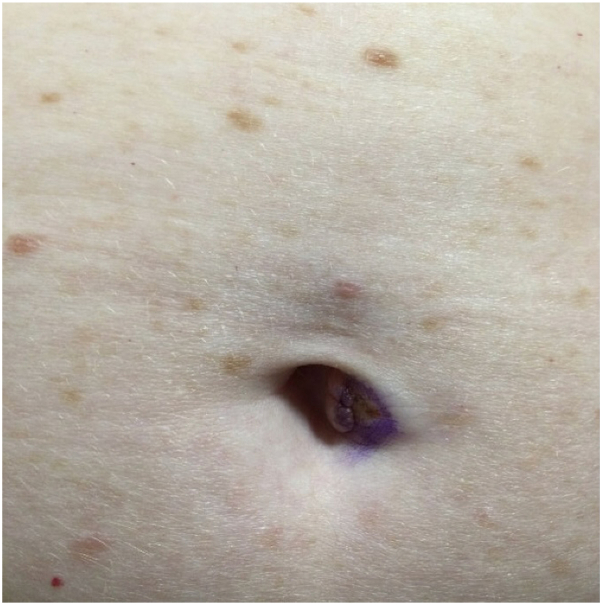
Pedunculated flesh-colored to slightly *pink papule* with a focal area of *dark brown* pigmentation within the umbilicus.

A 58-year-old woman presented with a 7 mm pigmented lesion in her umbilicus that she reported had been there since childhood. It was never painful, pruritic, and there was no associated bleeding or drainage. The patient noticed a new dark brown spot develop within the lesion, so the decision was made to perform a biopsy (Fig 1).

**Fig 2:**
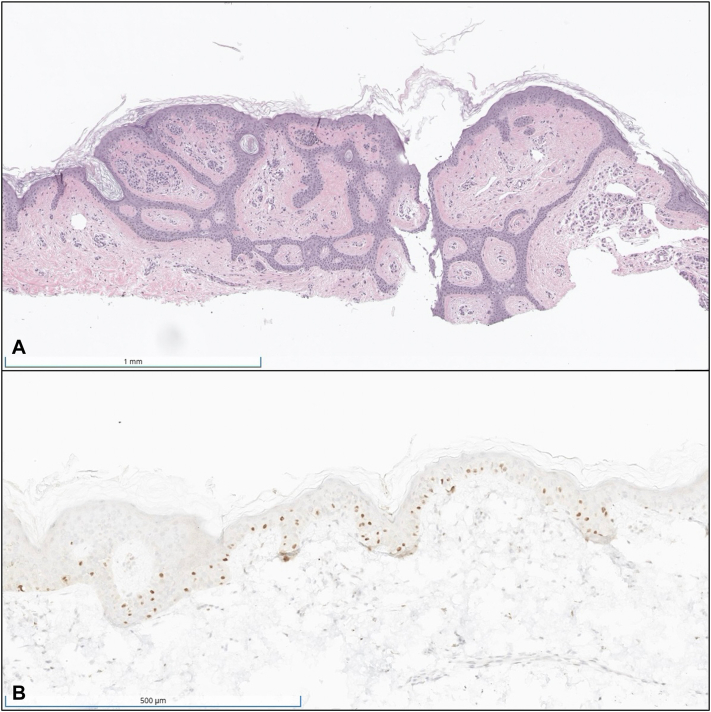
**A,** Shave biopsy specimen showing melanocytes in variably sized and shaped nests within the epidermis and dermis (H&E). **B,** PRAME positive staining highlighting the upward migration of melanocytes into the epidermis (PRAME).

H&E revealed a somewhat irregularly nested lesion within the epidermis and dermis, with subtle upward migration melanocytes in the epidermis (Fig 2).

**Fig 3:**
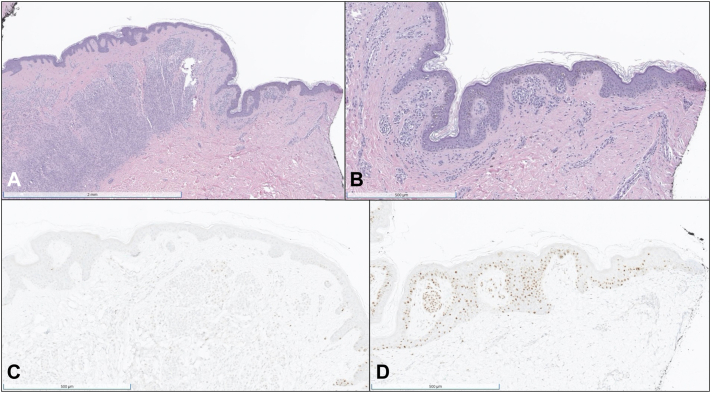
**A,** Excised specimen showing nested melanocytes primarily in the dermis (H&E). **B,** Detail of nested melanocytes and upward migration of melanocytes within the epidermis corresponding to focus of PRAME positivity at the margin of excised specimen (H&E). **C,** Majority of the excised specimen showing PRAME negativity (PRAME). **D,** Focus of PRAME positivity at the margin of excised specimen (PRAME).

PRAME and Melan-A immunohistochemical stains were performed with PRAME staining positive (3-4+) and Melan-A revealing melanocytes in nests and single units in a haphazard array within the epidermis with a foci of confluence. Castle MyPath Melanoma gene expression test resulted in a score of 1.2, indicating an intermediate gene expression profile that cannot exclude malignancy. A diagnosis of atypical melanocytic neoplasm was made based on these findings and complete removal of the lesion was recommended. The patient initially delayed surgical excision, but after additional encouragement regarding the importance of surgical removal, she underwent surgical excision of the tumor with a 2 mm margin under local anesthesia 3 months after the initial biopsy. PRAME and Melan-A staining of the excised specimen revealed PRAME positivity (4+) and Melan-A highlighted focal upward migration of melanocytes within the epidermis and marked uneven distribution of abnormal melanocytes in solitary units and nests within the epidermis (Fig 3).

Based on these findings, a diagnosis of melanoma in situ in association with a melanocytic nevus was made. Given peripheral margin positivity, the patient underwent re-excision with 5 mm margins under local anesthesia with no residual melanoma noted on pathology.

## Discussion

Melanomas are 1 of the most aggressive forms of skin cancer, which can arise from preexisting nevi or develop as a new cutaneous lesion. Primary cutaneous malignant melanomas involving the umbilicus are very rare, with only 46 cases being reported in the literature worldwide.^3^ Some cases developed from pre-existing nevi, while others de novo.

Umbilical melanomas are unique in their anatomical location. After birth, the umbilical cord develops into a scar that connects the peritoneum to abdominopelvic organs. The umbilicus also acts as an autonomous site for lymph drainage. This complex anatomy and close proximity of various abdominopelvic structures can provide an opportunity for primary umbilical melanoma to extend and metastasize. Thus, wide local excision of umbilical tumors requires an approach that reaches the peritoneum below the umbilicus and up to remaining muscular fascia.^4^ Risks of incomplete excision are local recurrence and metastatic spread.^2^ Sentinel lymph node biopsy should also be considered due to these complex connections to the lymphatic system.^4^ Dynamic and static lymphoscintigraphy with single-photon emission tomography/computed tomography can be used to identify all involved sentinel lymph nodes.^5^ Of the reported cases of umbilical melanoma for which treatment is known, wide local excision was the only surgical treatment attempted, with no cases reporting the use of Mohs micrographic surgery.^6^ When performing biopsy or excision in the umbilicus, it is important to do so carefully. The umbilicus can be a site of herniation due to the weakness of the abdominal wall at that area, placing visceral organs closer to the skin surface.

Early detection and monitoring of benign-appearing lesions in the umbilicus is important, especially when they exhibit changes over time. Patients with umbilical nevi should be advised to perform self-examinations for any changes in appearance or symptoms. These changes include color (like seen in this case), size, pain/pruritus, and bleeding. Presence of these changes should prompt early excisional biopsy to rule out potential malignant transformation. Cases of umbilical melanomas can result in metastatic disease within 7 months without treatment or even death from disease within 8 months from recurrence after wide local excision and regional lymphadenopathy.^6^ Therefore, it is important for patients to receive timely diagnosis and treatment, and then be scheduled for routine clinical monitoring to ensure no future complications arise.

This case highlights the rapid progression that umbilical melanomas can exhibit, and the importance of patient vigilance in monitoring for changes and physician encouragement for appropriate treatment and follow-up.

## Conflicts of interest

None disclosed.
